# Chloroform—Internally and Externally

**Published:** 1854-01

**Authors:** R. L. Madison

**Affiliations:** Petersburg, Va.


### *Chloroform—Its internal and external Uses.*

BY R. L. MADISON, M. D., PETERSBURG, VA.

The use of this powerful anæsthetic has become widely popular with the medical profession, and richly does it deserve its popularity. Having passed the ordeal of rigid experiment and enlarged experience, it now ranks *first* among those remedies which science, with lavish hand, has kindly furnished for our relief, while millions are ready, with willing lips, to testify to its delightful and lethean influence, and to herald its praises as a mighty boon to suffering humanity. If to afford immunity from pain, to impart relief to agonized human nature, be *one* among the many glorious objects of the physician's mission upon earth, surely the possession of this remedial agent alone were sufficient to yield the blessings of comparative comfort to half the "ills that flesh is heir to."

It is not my purpose, in the present article, to speak of the anæthetic virtues of chloroform, because they are too well known and appreciated by the profession to need further commendation or support, but simply, through the medium of your valuable journal, to invite attention more directly to some other uses to which it can be, and has been, applied with the same success, and with equal certainty, as by inhalation.

I allude to its *external* application and *internal* administration in cases of exalted or perverted nervous action, inducing spasm whether *tonic* or *clonic*, as exemplified in traumatic or idiopathic tetanus, delirium tremens, spasmodic colic, &c., &c.

Without further preface I will proceed, by way of illustration, to recite a few cases of recent occurrence, and let them speak for themselves.

Case I.—A little negro about two years of age, belonging to Mr. R———, of Dinwiddie county, was bitten on the heel, by a rat. This attracted very little attention until the afternoon of the third or fourth day, when the child was seized with a violent convulsion, followed, after an interval of an hour, by another of still greater violence. Being on a visit to a neighboring house, I was called in before the termination of the second spasm. He had been immersed in warm water and an emetic administered, which relieved his stomach of some well digested food. On enquiry whether he had received any external injury, his mother replied, "nothing except the bite of a rat." An examination of the heel revealed a punctured wound, swollen and tender to the touch, the inflammation extending some up the limb. The constitutional disturbance, however, was not sufficiently great to develope much fever. I expressed my fears to Mr. R——— of the tetanic nature of the spasms, and explained the danger to be apprehended from them.

*Treatment.*—Hot bath as often as the spasms should occur. Ley poultice to the heel, to be changed every hour or two, and the following liniment: ℞ Chloroform and ol. olivæ, aa ℥ij; spts. ammoniæ fort. ℥ss. M. To be rubbed well on the whole length of the spine every half hour until bedtime. Its use to be resumed during the night, if necessary.

October 4, 10 o'clock A. M.—One spasm had occurred, accompanied by violent rigidity of the muscles of the forearm and those of mastication, soon after the rubbing commenced. There were occasional nervous twitchings during the night, but otherwise slept composedly. Has no fever; pain and inflammation in the heel diminished. Continued the poultice, with spinal frictions every two hours during the day, and prescribed, ℞ Hyd. chlorid. mit., grs. iv.; pulv. ipecac, gr. i. M.

October 5, 10½ A. M.—Has had no more spasms; medicine acted once; appears quite lively. Ordered the liniment to be continued three times a day. The punctured wound rapidly healed, and the child has since remained in excellent health.

Case II.—J. C. young man, aged 28. Sanguine temperament and plethoric habit; had been sick with bilious colic for three or four days, and was convalescing; was suddenly seized (Oct. 15, 1 o'clock, P. M.) with a severe convulsion of delirium tremens, consequent upon excesses of the previous week, and the antiphlogistic treatment resorted to in his case. The convulsion left him in a partially comatose condition, with some heat about the head; pulse 120, small and irritable. Twenty minutes afterwards he had another spasm. I did not like to employ the usual remedies in this case, not only on account of the pre-existing biliary derangement, but also because of the cephalic symptoms. I therefore determined to use chloroform externally. With this view a liniment was ordered, composed of equal parts of chloroform and sweet oil, which was directed to be rubbed well upon the whole length of the spine every half hour. About three o'clock Mr. C. had another spasm of short duration. After this his pulse gradually diminished in frequency, and increased in volume and softness. From 5 to 6 o'clock had a refreshing nap, and awoke with a clear intellect.

October 16, 9 o'clock P. M.—Slept but little; is now very nervous and restless; mind inclined to wander; muscles tremulous. Ordered an aperient of calomel and bicarbonate of soda, with a continuation of the spinal frictions, which by evening entirely allayed all the abnormal symptoms.

Oct. 17.—Dismissed as not requiring further medical aid. No anodynes were given internally.

These cases are interesting as illustrative of the sedative and anodyne effects of chloroform where endermically applied, unattended with any possible dangerous results, and consequently obnoxious to no objections. These effects are further proven by the benefit derivable from the external use of chloroform in cases of simple neuralgia, muscular rheumatism, cramp, &c., &c., in many instances relief being immediate.

CASE III.—J. T., aged 25; laboring under mania potu.

Oct. 33.—Delirium constant, though of a low and muttering character; morbidly timid; pulse feeble and rapid; great enlargement of the liver, which is tender on pressure and percussion; tongue red and dry; complete anorexia and insomnia; extremities cold. He has been intemperate for years.

*Treatment.*—Sinapisms to the right hypochondrium and inferior extremities, and the following pill: ℞. Quinæ sulph., g. camphoræ aa., grs. xii; hyd. chlorid. mit., grs. x; pulv. opii., grs. xij. M. Syp. qs. F. in pil. No. xxiv. S. Two every two hours until bedtime.

Oct. 24, 9 A. M —A little better. Had rise of fever towards evening, with active delirium nearly all night. Has still considerable thirst, and complains much of his liver. Tongue covered with a dark fur, but moister than yesterday. Pulse 98 and feeble. Directed the pills to be continued once every three hours until 4 o'clock P. M., when he took the following draught: ℞. Quiniæ sulph., grs. xv; aq. camph., ℥ss.; elix., opii. ʒiss. M.

Oct. 25, 9½ P. M.—Rested somewhat better; delirum less active during the night; mind clear at intervals; gums a little tender; skin moist; pulse about 90, and more developed; anorexia and jactitation continue.

I ordered an ounce of castor oil; and at 5 o'clock P. M., he took ℞. Chloroform, ʒi.; aq. camph., ℥iss. M.

Oct. 25, A. M.—Says he feels a great deal better; slept well for the first time in a week; feels hungry; pulse full and soft; skin and tongue moist; the oil has acted twice.

After this he remained calm and rational, but I doubt whether his health will ever be fully restored, his system being completely shattered by long continued dissipation.

CASE IV.—R. H. W., æt about 30. Has had several attacks of arthritic rheumatism within the last eighteen months. Is suffering now (Oct. 26) from rheumatism attacking the muscular coat of the small intestines. Complains of great and incessant pain, constipation, considerable fever, furred tongue, loss of appetite, &c.

*Treatment.*—Hot emolient cataplasms to the abdomen; v. s. ℥xvi.; and R. Hyd. chlorid. mit., grs. x.; morphiæ muriat, gr. $\frac{1}{8}$; syp. qs. F. in pil. No. i.

Oct. 27, 9 A. M.—No better; did not sleep at all; pain at times intense, aggravated by large collections of wind in the ascending and transverse colon; urine very high colored; pulse jerking. Ordered a stimulating enema; continued the stimulating poultices to the abdomen; and gave: ℞. Ammoniæ phosphat. ʒiij; aq. font. ℥v; syp. aurat., cort. spts. ammonia aromat., aa. ℥ss. M. S. Tablespoonful every three hours in half a glass of water.

Oct. 28, 10 A. M.—Slept very little last night; pain still continues; enema acted well; no appetite, yet the tongue is clean and moist, the urine clear and the pulse nearly natural. The continued character of the pain and the inability to sleep induced me to prescribe chloroform internally. Accordingly, at 7 P. M., he took ℞. Chloroform, ʒij.; aq. camph., ℥ij. M. This draught produced sound and refreshing sleep for three or four hours, after which he was wakeful and free from pain. This morning (Oct. 29) says he feels almost entirely well; convalescence rapid.

There are very many cases in which it is desirable to administer an anodyne, and equally desirable not to produce constipation, or to suspend the peristaltic action of the intestines. In such cases chloroform, in my experience, is an admirable remedy, not only calming the nervous system and promptly inducing sleep, but creating no inertia of the digestive organs, and causing no vascular determination to the brain. There are persons also, in every community, who cannot take opium, or any of its preparations. These, I should think, might take chloroform with impunity, although I have had no opportunity as yet for testing the experiment.—*Stethoscope.*

